# Baicalein as a Potential Inhibitor against BACE1 and AChE: Mechanistic Comprehension through In Vitro and Computational Approaches

**DOI:** 10.3390/nu11112694

**Published:** 2019-11-07

**Authors:** Jin Han, Yeongseon Ji, Kumju Youn, GyuTae Lim, Jinhyuk Lee, Dong Hyun Kim, Mira Jun

**Affiliations:** 1 Department of Food Science and Nutrition, College of Health Sciences, Dong-A University, Busan 49315, Korea; lrene4079@naver.com (J.H.); jysun3410@naver.com (Y.J.); kjyoun@dau.ac.kr (K.Y.); 2 Center for Silver-Targeted Biomaterials, Brain Busan 21 Plus Program, Graduate School, Dong-A University, Busan 49315, Korea; 3 Korean Bioinformation Center, Korea Research Institute of Bioscience and Biotechnology (KRIBB), Daejeon 34141, Korea; gyutae@kribb.re.kr (G.L.); jinhyuk@kribb.re.kr (J.L.); 4 Department of Bioinformatics, KRIBB School of Bioscience, Korea University of Sciences and Technology, Daejeon 34113, Korea; 5 Department of Medicinal Biotechnology, College of Health Sciences, Dong-A University, Busan 49315, Korea; mose79@dau.ac.kr; 6 Institute of Convergence Bio-Health, Dong-A University, Busan 49315, Korea

**Keywords:** Alzheimer’s disease, Aβ, baicalein, BACE1, docking analysis, Lipinski’s rules

## Abstract

One of the major neurodegenerative features of Alzheimer’s disease (AD) is the presence of neurotoxic amyloid plaques composed of amyloid beta peptide (Aβ). β-Secretase (BACE1) and acetylcholinesterase (AChE), which promote Aβ fibril formation, have become attractive therapeutic targets for AD. P-glycoprotein (P-gp), the major efflux pump of the blood-brain barrier (BBB), plays a critical role in limiting therapeutic molecules. In pursuit of discovering a natural anti-AD candidate, the bioactivity, physicochemical, drug-likeness, and molecular docking properties of baicalein, a major compound from *Scutellaria baicalensis*, was investigated. Baicalein exhibited strong BACE1 and AChE inhibitory properties (IC_50_ 23.71 ± 1.91 µM and 45.95 ± 3.44 µM, respectively) and reacted in non-competitive and competitive manners with substrates, respectively. in Silico docking analysis was in full agreement with the in vitro results, demonstrating that the compound exhibited powerful binding interaction with target enzymes. Particularly, three continuous hydroxyl groups on the A ring demonstrated strong H-bond binding properties. It is also noteworthy that baicalein complied with all requirements of Lipinski’s rule of five by its optimal physicochemical properties for both oral bioavailability and blood–brain barrier permeability. Overall, the present study strongly demonstrated the possibility of baicalein having in vivo pharmacological efficacy for specific targets in the prevention and/or treatment of AD.

## 1. Introduction

Alzheimer’s disease (AD) is a progressive neurodegenerative disorder that slowly weakens memory, cognition, and daily functions. The predominant neuropathological hallmarks of AD are neurofibrillary tangles and amyloid plaques in the brain. These are characterized by the presence of intracellular hyperphosphorylated tau protein and extracellular deposits of aggregated amyloid-peptide (Aβ), respectively. There is currently no precise mechanism available to prevent and/or cure AD. However, because the deposition of Aβ is a critical feature and the first initiating pathological event of AD, a large body of evidence has strongly supported that Aβ theory is a central strategy for developing effective treatments for AD [1].

Aβ is derived from the sequential cleavage of amyloid precursor protein (APP) through two-step proteolytic processing. β-Secretase (BACE1) first initiates the cleavage at APP, and γ-secretase subsequently cleaves this fragment to releasing Aβ in 37–43 amino acid sequences. BACE1 plays a critical role in the rate-limiting steps of Aβ production, the inhibition of this enzyme became widely acknowledged to regulate the production of Aβ [2].

Oral administration of a potent and selective BACE1 inhibitor effectively lowered β-cleavage of APP and Aβ production in APP transgenic mice [3]. Furthermore, animals lacking the BACE1 gene developed normally and showed no critical biochemical or behavioral problems, suggesting that deletion of BACE1 activity results in no major adverse effects [4]. In contrast, γ-secretase participates in numerous transmembrane protein cleavages and is involved in the processing of Notch cleavage catalysis, acting as an embryonic development regulator [5]. Several studies have demonstrated that γ-secretase inhibition can cause neurotoxicity in vivo, supporting BACE1 inhibition being the most effective anti-amyloid target in Aβ production-related AD [6].

Cholinesterases, namely acetyl- and butyrylcholinesterase (AChE and BChE, respectively), break down acetylcholine (ACh) into choline and acetate through hydrolysis in the synaptic clefts, resulting in reduced signal transmission. Numerous studies have demonstrated that AChE not only accelerates Aβ aggregation and amyloid fibril plaque formation, but also influences the conformational and biochemical changes of Aβ. These changes accelerate Aβ fibrils by forming stable AChE-Aβ complexes [7,8]. Furthermore, AChE-Aβ complexes triggered more enhanced neurotoxicity than Aβ alone [9]. As a result, dual enzymatic inhibition of BACE1 and AChE, as well as Aβ aggregation, can be proposed as a promising and preventive route against AD.

The blood–brain barrier (BBB) provides an enormous blockade between the blood and parenchyma cells of the brain. The ability to penetrate the BBB is therefore essential for anti-AD agents. P-glycoprotein (P-gp), an efflux transporter located in the endothelial cell membrane of the BBB, makes inflow of diverse molecules from the central nervous system (CNS) difficult, suggesting that the P-gp variation is related to the onset of neurodegenerative diseases such as AD [10]. Since P-gp plays a crucial role in predicting the possibility of passing the BBB, the inhibition of this efflux pump can prevent P-gp-mediated molecule efflux and assist in CNS entry.

*Scutellaria baicalensis* (*S. baicalensis*) has been widely used in traditional medicine in China and Korea [11]. Baicalein, the major bioactive compound of *S. baicalensis*, has diverse biological activities, such as antioxidant, anti-thrombotic, and anti-inflammatory effects [12]. Furthermore, in regards to neuroprotection, the compound has been reported to reduce the production of inflammatory cytokines and attenuate Aβ-stimulated apoptosis in the rat cerebral cortex [13,14]. Recently, behavioral dysfunction in the AlCl_3_-induced AD mouse model was improved by baicalein treatment [15]. Two important recent discoveries have demonstrated that baicalein has the ability to cross the BBB in about half an hour after administration (30 mg/kg^−1^), and was partially identified in the rat brain after oral administration (30 mg/kg^−1^) [16,17].

Although several studies have reported the neuroprotective effects of baicalein, there is currently no detailed research regarding the mechanisms of molecular interaction between baicalein and AD-related enzymes, such as BACE1 and AChE. Thus, in the present study, to identify the potential of baicalein as a therapeutic candidate in AD, an in vitro study was conducted for drug-likeness prediction and an in silico docking simulation was performed.

## 2. Materials and Methods

### 2.1. Chemicals and Reagent

Baicalein (≥98% purity) was obtained from Chemfaces (Wuhan, China). Resveratrol (>99% purity), galantamine, and 5,5′-dithiobis-(2 nitrobenzoic acid) (DTNB) were obtained from Sigma-Aldrich (St. Louis, MO, USA). α-Secretase (TACE) and its substrate were purchased from R&D Systems (Minneapolis, MN, USA). The BACE1 FRET assay kit was purchased from Invitrogen (Pan Vera Co., Madison, WI, USA). AChE, chymotrypsin, trypsin, and elastase, as well as their substrates, were purchased from Sigma-Aldrich (St. Louis, MO, USA).

### 2.2. Enzymatic Assay for Biological Evaluation

BACE1, AChE, TACE, trypsin, chymotrypsin, and elastase assays were carried out according to previously described methods [18]. Fluorometric assays, reaction mixtures containing human recombinant BACE1 (1.0 U/mL), the substrate (Rh-EVNLDAEFK-Quencher in 50 mM ammonium bicarbonate), and baicalein dissolved in an assay buffer (50 mM sodium acetate, pH 4.5) were incubated in darkness for 60 min at 25 °C in 384-well plates. The increase in fluorescence intensity produced by substrate hydrolysis was observed on a fluorescence microplate reader with excitation and emission wavelengths of 545 and 590 nm, respectively. The inhibition ratio was obtained using the following equation:

Inhibition (%) = [1 − (S − S0)/(C − C0)] × 100

where C is the fluorescence of control (enzyme, assay buffer, and substrate) after 60 min of incubation, C0 is the fluorescence of control at time 0, S is the fluorescence of tested samples (enzyme, sample solution, and substrate) after 60 min of incubation, and S0 is the fluorescence of the tested samples at time 0.

A human recombinant TACE (0.1 ppm in 25 mM Tris buffer), the substrate (APP peptide YEVHHQKLV using EDANS/DABCYL), and baicalein were dissolved in an assay buffer and then combined and incubated for 60 min in the dark at 25 °C. The increase in fluorescence intensity produced by substrate hydrolysis was observed on a fluorescence microplate reader with excitation and emission wavelengths of 320 and 405 nm, respectively.

The colorimetric assays of AChE, trypsin, chymotrypsin, and elastase were carried out using acetylthiocholine iodide, *N*-benzoyl-L-Tyr-pNA, *N*-benzoyl-L-Arg-pNA, and *N*-succinyl-Ala-Ala-Ala-pNA as substrates, respectively. The hydrolysis of AChE was monitored according to the formation of yellow 5-thio-2-nitrobenzoate anions at 405 nm for 15 min, which were produced by the reaction of DTNB with thiocholine released from ACh. All reactions were performed in 96-well plates in triplicate and recorded using a microplate spectrophotometer.

For trypsin, chymotrypsin, and elastase assays, enzyme, Tris-HCl buffer (0.05 M, in 0.02 M CaCl_2_, pH 8.2), and baicalein were incubated for 10 min at 25 °C; then, substrate was added for 30 min at 37 °C. The absorbance was recorded at 410 nm. The inhibition ratio was obtained using the following equation:

Inhibition (%) = {[1 − (A − B)]/control} × 100

where A was the absorbance of the control (enzyme, assay buffer, and substrate) after 60 min of incubation, and B was the absorbance of tested sample (assay buffer and sample solution) after 60 min of incubation.

### 2.3. Kinetic Studies of Baicalein on BACE1 and AChE

Dixon and Lineweaver–Burk plots were conducted to examine the kinetic mechanisms of baicalein against BACE1 and AChE with varying concentrations of substrate and inhibitors. The inhibitory constant (Ki) was obtained by interpretation of the Dixon plot, and maximum reaction velocity (Vmax) and Michaelis–Menten constant (Km) values were determined by a Lineweaver–Burk plot, using the initial velocities obtained over a different substrate concentration ranges. The kinetic parameters were determined using Enzyme Kinetic^TM^ module of SigmaPlot^TM^ version 12.3 (Systat Software, Inc., San Jose, CA, USA).

### 2.4. In Silico Molecular Docking Studies and Drug-Likeness Prediction

Molecular docking analysis was performed by the AutoDock Vina program to investigate structural complexes of BACE1, AChE, and P-gp with baicalein. For docking analysis, the 3D crystal structures of BACE1, AChE and P-gp were obtained from the Protein Data Bank (PDB ID: 2WJO, 4PQE and 3G60, respectively), and the structure of baicalein was prepared from the Pubchem database (CID 5281605). The Autodock Vina parameters were employed to create a grid box of size 30 Å × 30 Å × 30 Å. All docking structures were ranked and categorized by the lowest energy, and the best poses were selected [19].

Considering the bioavailability, drug-likeness prediction was performed to assess the possibility for a molecule to become an oral drug using SwissADME (http://www.swissadme.ch).

### 2.5. Statistical Analysis

All results are expressed as the mean ± standard deviation (SD) and analyses were performed in triplicate. Significant difference was measured by Duncan’s multiple range tests using Statistical Analysis System (SAS) version 9.3 (SAS Institute, Cary, NC, USA).

## 3. Results and Discussion

### 3.1. In Vitro Inhibitory Activity of Baicalein against BACE1 and AChE

**Figure 1:**
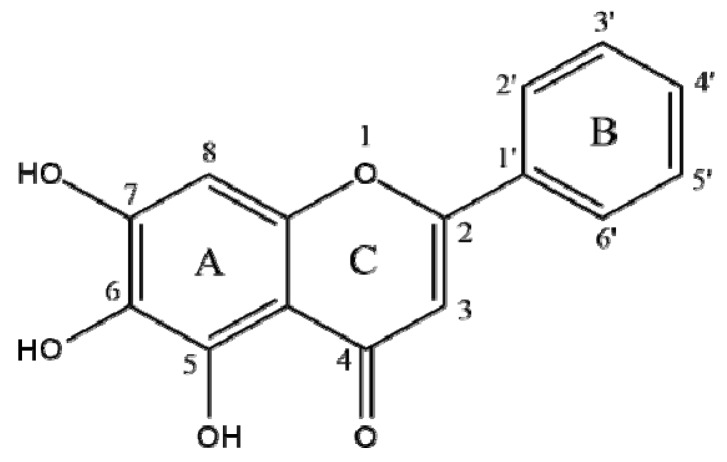
The chemical structure of baicalein.

The chemical structure of baicalein (5,6,7-trihydroxyflavone) is shown in Figure 1. To determine the anti-AD potential of baicalein, its inhibitory activity against BACE1 and AChE was evaluated (Table 1). The compound strongly inhibited BACE1 in a dose-dependent manner (*p* < 0.001) with an IC_50_ value of 23.71 ± 1.91 µM.

In a previous antioxidant study, baicalein exhibited the most powerful antioxidant effect in chemical scavenging, as well as the most potent cellular antioxidant ability among the flavonoid components of *S. baicalensis* [20]. Chrysin is a flavone structurally similar to baicalein, with two hydroxyl groups at C-5 and -7 on the A ring. This flavone showed much weaker antioxidant ability relative to that of baicalein. It can be assumed that the presence of three continuous hydroxyl groups on the A ring improves its hydrogen or electron donating potential, as well as its BACE1 inhibitory activity. In addition, because oxidative stress abnormally increases BACE1 gene expression and leads to Aβ accumulation, the excellent antioxidant activity of baicalein is expected to influence BACE1 inhibition through delaying or reducing oxidative damage [21]. Apigenin (4′,5,7-trihydroxyflavone) is found in a wide variety of plants and is structurally similar to baicalein, save for the repositioning of one hydroxyl group (C-6 to C-4′). Apigenin was reported to exhibit poor BACE1 inhibitory activity (IC_50_ > 100 µM), assuming that a hydroxyl group at C-6 of baicalein provides a partial inhibition of BACE1 [22].

In addition, baicalein dose-dependently and significantly (*p* < 0.001) suppressed AChE with an IC_50_ value of 45.95 ± 3.44 µM. Although baicalein showed somewhat weaker inhibition of AChE compared with galantamine, a well-known positive control with an IC_50_ value of 1.30 ± 0.01 µM, it is still considered as potential bioactive compound that could prevent both Aβ aggregation and amyloid fibril plaque formation.

### 3.2. Enzyme Kinetic Analysis of BACE1 and AChE Inhibition

**Figure 2:**
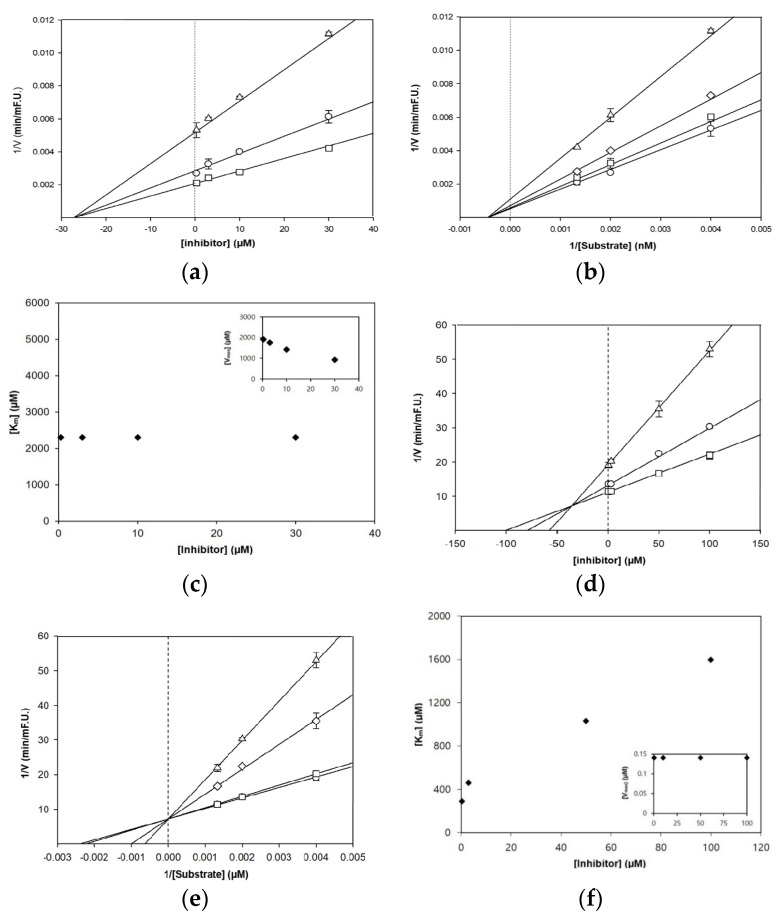
Dixon and Lineweaver–Burk plots for the inhibition of BACE1 (**a**–**c**) and AChE (**d**–**f**) by baicalein. Dixon plot of BACE1 inhibition by baicalein in the presence of different substrate concentrations: 250 nM (∆), 500 nM (○), and 750 nM (□) (**a**). Dixon plot of AChE inhibition by baicalein in the presence of different substrate concentrations: 250 µM (∆), 500 µM (○), and 750 µM (□) (**d**). Lineweaver–Burk plot was analyzed in the presence of different inhibitor concentrations: 0.3 µM (○), 3 µM (□), 10 µM (◊), and 30 µM (∆) (**b**); and 0.3 µM (○), 3 µM (□), 50 µM (◊), and 100 µM (∆) (**e**). Km values as a function of the concentrations of baicalein (Inset) Dependence of the values of Vmax on the concentration of baicalein (**c**,**f**).

In an attempt to analyze the mechanism of enzyme activity and the inhibitor binding sites, kinetic analysis of BACE1 and AChE for baicalein was performed (Table 1 and Figure 2). The Dixon plot indicates that baicalein acts as a non-competitive BACE1 inhibitor, binding either to another regulatory site or to subsites of BACE1 with a Ki value of 27.6 µM. Lower Ki values indicate tighter enzyme binding and more effective inhibitory activity. Baicalein with a lower Ki value could be an effective BACE1 inhibitor. This value is typically in the range of 4.4–82.6 µM for natural flavonoids [18,19,23].

Interestingly, the kinetic results showed that baicalein inhibited both BACE1 and AChE in different inhibition modes. Lineweaver–Burk reciprocal plots of baicalein against AChE displayed unchanged Vmax and increased Km values with an increase in inhibitor concentration. This indicates that baicalein was fitted well to the competitive inhibitor, binding tightly at a catalytic site of AChE instead of at other allosteric sites, with a Ki value of 35.9 µM.

### 3.3. Selectivity of Baicalein against BACE1

In the non-amyloidogenic pathway, α-secretase (TACE) generates a soluble neurotrophic fragment (sAPPα), preventing Aβ fibril formation by BACE1. In addition, it is well established that serine proteases are important for various normal physiological processes, such as food digestion, blood coagulation, wound healing, and inflammatory responses. This indicates that selective and specific inhibition of targeted enzymes, without affecting TACE and other serine proteases, is important. As shown in Table 2, baicalein had no significant inhibitory activity against TACE and other serine proteases, such as trypsin, chymotrypsin, and elastase up to 100 µM. This demonstrated that baicalein is a selective and specific inhibitor against BACE1.

### 3.4. In Silico Docking Simulation of Baicalein and Its Drug-Likeness Prediction

**Figure 3:**
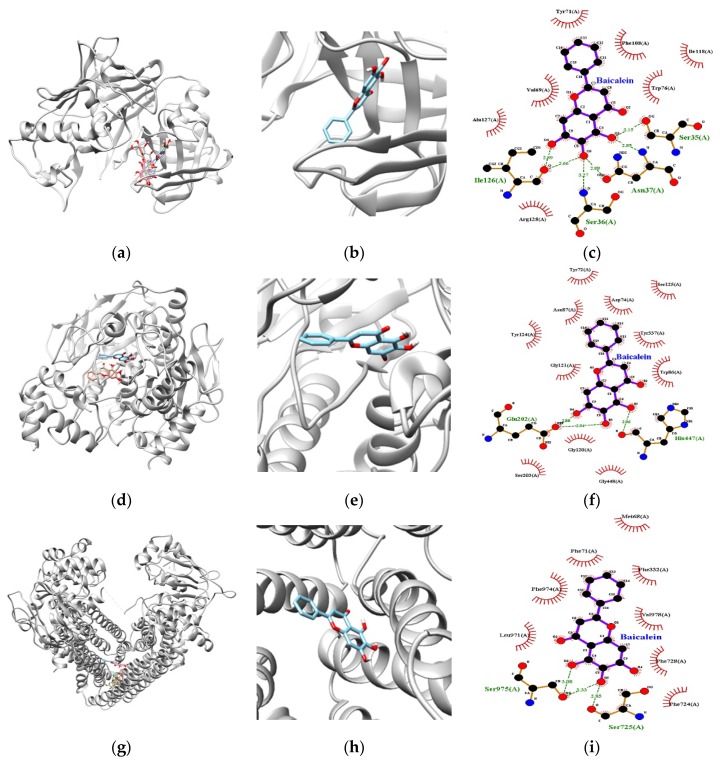
Docking simulation of the structure for BACE1, AChE, and P-gp with baicalein. The best docking poses between BACE1, AChE, and P-gp and baicalein (**a**,**d**,**g**). View of the binding site magnified from baicalein (**b**,**e**,**h**). 2D ligand interaction diagrams of BACE1, AChE, and P-gp inhibition by baicalein (**c**,**f**,**i**). Green dotted lines indicate H-bonds. Non-ligand residues involved in hydrophobic interactions are marked with open spokes. Atoms are colored as follows; carbon atoms in black, nitrogen atoms in blue, and oxygen atoms in red.

The in silico molecular interaction of BACE1, AChE, and P-gp with baicalein is summarized in Table 3. According to the results of docking simulation in Figure 3a–c, the baicalein-BACE1 complex exhibited negative binding energy (−8.60 kcal/mol), suggesting that the compound is a high-affinity BACE1 inhibitor able to bind strongly with the enzyme. Although baicalein is non-competitive BACE1 inhibitor that docks into a non-catalytic region, such as Ser36, Asn37, and Ile26 residues of BACE1, it also binds to the catalytic region Ser35. The catalytic domain of BACE harbors two aspartic protease active site motifs of the sequence DTGS and DSGT that come together to form the active site of the enzyme. Ser35 is a part of the DTGS active site motif of BACE1 and it was found to interact with a water molecule critical for BACE1 hydrolytic activity [2,24]. Moreover, six hydrogen bonds were observed between the oxygen groups of Ser35, Ser36, Asn37, and Ile26 and the three hydroxyl groups of baicalein, with bonding distances of 3.15, 3.27, 2.85, and 2.88 and 2.66 and 2.89 Å, respectively. Interestingly, the hydroxyl moiety at C-6 on the A ring of baicalein simultaneously acted as a hydrogen donor to Ile126 and Asn37 residues and an acceptor to Ser36. In contrast, the oxygen atom at C-5 on the A ring accepted the hydrogen from the Asn37 and Ser35 residues, and the oxygen atom at C-7 on the A ring donated a hydrogen to Ile126. The presence of an H-bond donor or acceptor on the A ring may therefore affect the potential inhibition of BACE1 in the biological evaluation results of baicalein.

As shown in Figure 3d–f, baicalein was revealed to be active against AChE with a lowest energy of −8.7 kcal/mol. The catalytic site of AChE is located at the bottom of a narrow gorge known as the catalytic triad, composed of Ser203, Glu334, and His447. The hydroxyl group at C-5 on the A ring produced close binding with one of the catalytic triads (His447) through a H-bond (bonding distance 2.94 Å). In addition, the hydroxyl groups at C-6 and C-7 on the A ring of baicalein bound to the oxygen atom of Glu202, with a H-bonding distance of 2.88 and 2.94 Å, respectively. A peripheral site comprised of aromatic side chains including Tyr72, Asp74, Tyr124, Trp286, Phe295, Phe297, Tyr337, and Tyr341 contributes to the catalytic efficiency of AChE [25]. A total of 11 hydrophobic interactions were exhibited by Tyr72, Asp74, Trp86, Asn87, Gly120, Gly121, Tyr124, Ser125, Ser203, Tyr337, and Gly448. Overall, these docking results were in agreement with our in vitro biological experimental results, demonstrating that baicalein is a crucial BACE1 inhibitor connected with BACE1 by stronger H-bonds than with AChE.

Furthermore, for the effective prevention and/or treatment of AD, docking simulation between P-gp and baicalein was performed to predict the possible effluence system. As shown in Table 3 and Figure 3g–i, the lowest binding energy between baicalein and P-gp residues was −8.4 kcal/mol. Three H-bonds were observed in the baicalein-P-gp complex, and Ser975 and Ser725 residues participated in this interaction. As with the BACE1 docking study, the oxygen atom of baicalein at C-6 of the A ring simultaneously acted as a hydrogen donor to Ser725 residues and an acceptor to Ser975, with bonding distances of 2.85 and 3.33 Å, respectively. In addition, the hydroxyl group at C-5 on the A ring formed one H-bond with Ser975, with a bond distance of 3.08 Å. Aside from these H-bonds, other residues such as Phe724, Phe728, Val978, Phe332, Phe71, Phe974, and Leu971 were found to have van der Waals interactions.

Lipinski’s criteria, also known as the “rule of five”, is generally considered a drug-likeness test for the evaluation of oral availability for compounds. It states that poor absorption or permeability of a compound occurs when there are more than 5 H-bond donors, more than 10 H-bond acceptors, more than 5 calculated Log P, and the molecular weight is higher than 500 Da [26]. Additionally, good bioavailability is more likely for compounds with a total polar surface area (TPSA) of ≤140 Å and ≤10 rotatable bonds (nrotb). It is noteworthy that baicalein complied with all requirements of Lipinski’s rule of five for both oral bioavailability and BBB permeability, as listed in Table 4.

Several previous studies have demonstrated that baicalein possesses antioxidant activity by reducing Aβ-induced neurotoxicity in PC12 cells, thus protecting rat cortical cells [13,27]. The compound prevented H_2_O_2_-induced apoptosis and restored mitochondrial dysfunction in PC12 cells [28]. In addition, in vivo studies have demonstrated that baicalein significantly improves the cognitive performance of Tg2576 mice (10 mg/kg) and inhibits LOX and GSK3β activity, consequently preventing tau phosphorylation in APP/PS1 mice (80 mg/kg) [29,30].

Oral acute toxicity studies are needed to confirm a range of doses for compounds and to reveal the safety of clinical signs. Several studies demonstrated that baicalein expresses no signs of toxicity after 2.8 mg of single-dose administration, demonstrating safety for use with humans [31]. Another clinical trial demonstrated that baicalein is safe and well tolerated after multiple-dose oral administration (200, 400, and 800 mg) in healthy participants [32]. Even though natural BACE1 inhibitors such as baicalein possess relatively weaker BACE1 inhibitory properties compared with synthetic ones, they may be free of the side effects caused by excessive BACE1 inhibition.

The BBB not only protects the brain from the entry of potentially toxic substances, but also prevents the passage of therapeutic agents required for treating disorders related to neurodegenerative diseases. Therefore, one important issue in successful anti-AD therapy is the ability of the molecules to cross the BBB. Studies have shown that certain substances with lipid-soluble molecules and a low molecular weight of below 400–600 Da are transported across the BBB [33]. It is of great interest that baicalein has been reported to cross the BBB, and has been found in the rat brain after oral administration of *S. baicalensis* extract [17].

To be considered a promising anti-AD substance, both oral bioavailability and the metabolic transformation of compounds are necessary. Baicalein is the main bioactive constituent of S. baicalensis and is metabolized to baicalin, 7-O-glucuronide of baicalein, following intake by various animals and humans [34,35,36]. On the other hand, another study found that when baicalein was administered (30 and 60 mg/kg^−1^) through the femoral vein, intact baicalein was observed in rat plasma, the brain, and bile, and that this compound rapidly penetrated the BBB [16]. In addition, after oral administration of *S. baicalensis* extract (30 mg/kg^−1^), both baicalein and baicalin were detected in the rat brain, supporting the evidence that these two compounds could penetrate the BBB. Interestingly, the brain concentration of baicalein was greatly higher than that of baicalin. The brain-to-plasma ratio of baicalein was also twenty times higher than that of baicalin [17]. Although several studies have revealed that baicalein was converted to glucuronide form in vivo, baicalin as well as baicalein exhibited pharmacological efficacy, including antioxidant, anti-inflammatory, and neuroprotective properties. Baicalein fractionally crossed the BBB and reached the brain, indicating that baicalein could act as a natural anti-AD candidate [37]. Although further and more detailed studies need to be conducted, baicalein appears to be safe and exerts specific inhibitory properties against BACE1 and AChE, as well as P-gp inhibitory potential. It may therefore be considered a novel potential agent for preventing AD.

## 4. Conclusions

Our in vitro study demonstrated that baicalein is a selective and specific inhibitor against BACE1 and AChE in a non-competitive and a competitive mode, respectively. Moreover, an in silico docking simulation study revealed that baicalein possesses strong hydrogen bonding with several crucial amino acid residues in target enzymes when observing the lowest binding energies. It is expected that the presence of a H-bond at C-6 on the A ring greatly influenced the activity of baicalein. Although further and more detailed in vivo studies of baicalein need to be conducted, baicalein could be a beneficial candidate for the development of therapeutic and preventive agents of AD. This is demonstrated by its selective and specific BACE1 and AChE inhibition, as well as its strong binding affinity with P-gp for BBB permeability.

## Figures and Tables

**Table 1:** BACE1 and AChE inhibitory activities and kinetic analysis of baicalein.

Compounds	BACE1			AChE		
	IC_50_^1^	K_i_ Value ^2^	Inhibition Type ^3^	IC_50_	K_i_ Value	Inhibition Type
Baicalein	23.71 ± 1.91	27.6	Non-competitive	45.95 ± 3.44	35.9	Competitive
Resveratrol ^4^	15.04 ± 0.87					
Galantamine ^5^				1.30 ± 0.01		

^1^ The 50% inhibition concentration (IC_50_, µM) is expressed as mean ± standard deviation (SD) of triplicate experiments. ^2^ Inhibition constants (Ki, µM) were determined using a Dixon plot. ^3^ Inhibition modes were determined using Dixon and Lineweaver–Burk plots. ^4^ Resveratrol and ^5^ Galantamine were used as positive controls for the BACE1 and AChE assays, respectively.

**Table 2:** Inhibitory activities (%) ^1,2^ of baicalein against α-secretase (TACE) and other serine protease.

Baicalein (μM)	TACE	Trypsin	Chymotrypsin	Elastase
50	8.31 ± 0.89	3.40 ± 0.76	8.42 ± 0.76	10.29 ± 1.04
100	9.45 ± 0.71	−6.95 ± 0.87	4.48 ± 0.76	9.00 ± 0.45

^1^ The inhibition activity (%) of baicalein against TACE, trypsin, chymotrypsin, and elastase is expressed as mean ± SD of independent triplicate experiments. ^2^ Comparison of concentration level of baicalein is not significantly different at *p* < 0.05.

**Table 3:** Molecular interaction of BACE1, AChE, and P-glycoprotein (P-gp) active sites with its ligand (Baicalein).

Enzymes	Lowest Energy (Kcal/moL)	No. of H-Bond ^1^	Residues	Bond Distance (Å)	Van der Waals Interacting Residues
BACE1	−8.60	6	Ser35	3.15	Val69, Tyr71, Trp76, Phe108, Ile118, Ala127, Arg128
			Ser36	3.27	
			Asn37	2.85 and 2.88	
			Ile126	2.66 and 2.89	
AChE	−8.70	3	Glu202	2.88 and 2.94	Tyr72, Asp74, Trp86, Asn87, Gly120, Gly121, Tyr124, Ser125, Ser203, Tyr337, Gly448
			His447	2.96	
P-gp	−8.40	3	Ser975	3.08 and 3.33	Met68, Phe71, Phe332, Phe724, Phe728, Leu971, Phe974, Val978
			Ser725	2.85	

^1^ The number of hydrogen bonds from the enzyme-inhibitor complex were determined with the Autodock Vina.

**Table 4:** In Silico drug-likeness property through Lipinski’s rule.

Compounds	No. of Violations	MW (g/moL)	H-Bond Acceptor	H-Bond Donor	Log P_o/w_^1^	TPSA ^2^ (Å^2^)	No. of rotb ^3^
Baicalein	0	270.24	5	3	2.682	90.89	1

^1^ Log P_o/w_ octanol/water partition coefficient ^2^ TPSA topological polar surface area ^3^ No. of rotb number of rotatable bonds.
